# The oxidized phospholipid PGPC impairs endothelial function by promoting endothelial cell ferroptosis via FABP3

**DOI:** 10.1016/j.jlr.2024.100499

**Published:** 2024-01-11

**Authors:** Si Chen, Jian-Jun Gao, Yu-Jia Liu, Zhi-Wei Mo, Fang-Yuan Wu, Zuo-Jun Hu, Yue-Ming Peng, Xiao-Qin Zhang, Zhen-Sheng Ma, Ze-Long Liu, Jian-Yun Yan, Zhi-Jun Ou, Yan Li, Jing-Song Ou

**Affiliations:** 1 Division of Cardiac Surgery, Cardiovascular Diseases Institute, The First Affiliated Hospital, Sun Yat-sen University, Guangzhou, China; 2 National-Guangdong Joint Engineering Laboratory for Diagnosis and Treatment of Vascular Diseases, NHC Key Laboratory of Assisted Circulation and Vascular Diseases (Sun Yat-sen University), Key Laboratory of Assisted Circulation and Vascular Diseases, Chinese Academy of Medical Sciences, Guangdong Provincial Engineering and Technology Center for Diagnosis and Treatment of Vascular Diseases, Guangzhou, China; 3 Division of Hypertension and Vascular Diseases, Department of Cardiology, Cardiovascular Diseases Institute, The First Affiliated Hospital, Sun Yat-sen University, Guangzhou, China; 4 Division of Vascular Surgery, The First Affiliated Hospital, Sun Yat-sen University, Guangzhou, China; 5 Department of Cardiology, Laboratory of Heart Center, Heart Center, Zhujiang Hospital, Southern Medical University, Guangzhou, China; 6 Guangdong Provincial Key Laboratory of Cardiac Function and Microcirculation, Guangzhou, China; 7 Guangdong Provincial Biomedical Engineering Technology Research Center for Cardiovascular Disease, Guangzhou, China; 8 Guangdong Provincial Key Laboratory of Brain Function and Disease, Zhongshan School of Medicine, Sun Yat-sen University, Guangzhou, China

**Keywords:** Oxidized lipids, PGPC, Fatty acid binding protein-3, CD36, Endothelial function, Atherosclerosis

## Abstract

Ferroptosis is a novel cell death mechanism that is mediated by iron-dependent lipid peroxidation. It may be involved in atherosclerosis development. Products of phospholipid oxidation play a key role in atherosclerosis. 1-palmitoyl-2-glutaroyl-sn-glycero-3-phosphocholine (PGPC) is a phospholipid oxidation product present in atherosclerotic lesions. It remains unclear whether PGPC causes atherosclerosis by inducing endothelial cell ferroptosis. In this study, human umbilical vein endothelial cells (HUVECs) were treated with PGPC. Intracellular levels of ferrous iron, lipid peroxidation, superoxide anions (O_2_^•−^), and glutathione were detected, and expression of fatty acid binding protein-3 (FABP3), glutathione peroxidase 4 (GPX4), and CD36 were measured. Additionally, the mitochondrial membrane potential (MMP) was determined. Aortas from C57BL6 mice were isolated for vasodilation testing. Results showed that PGPC increased ferrous iron levels, the production of lipid peroxidation and O_2_^•−^, and FABP3 expression. However, PGPC inhibited the expression of GPX4 and glutathione production and destroyed normal MMP. These effects were also blocked by ferrostatin-1, an inhibitor of ferroptosis. FABP3 silencing significantly reversed the effect of PGPC. Furthermore, PGPC stimulated CD36 expression. Conversely, CD36 silencing reversed the effects of PGPC, including PGPC-induced FABP3 expression. Importantly, E06, a direct inhibitor of the oxidized 1-palmitoyl-2-arachidonoyl-phosphatidylcholine IgM natural antibody, inhibited the effects of PGPC. Finally, PGPC impaired endothelium-dependent vasodilation, ferrostatin-1 or FABP3 inhibitors inhibited this impairment. Our data demonstrate that PGPC impairs endothelial function by inducing endothelial cell ferroptosis through the CD36 receptor to increase FABP3 expression. Our findings provide new insights into the mechanisms of atherosclerosis and a therapeutic target for atherosclerosis.

Endothelial dysfunction is the early stage of atherosclerosis. A variety of oxidized phospholipids have been found in atherosclerotic plaque (1). A recent study demonstrated that a reduction in oxidized phospholipids inhibits atherosclerosis (2). We have previously reported that a component of oxidized phospholipid 1-palmitoyl-2-(5-ox-ovaleroyl)-sn-glycero-3-phosphocholine (POVPC) and 25–hydroxycholesterol found in atherosclerotic plaque can impair endothelial function, which may contribute to the development of atherosclerosis (3, 4, 5). 1-palmitoyl-2-glutaroyl-sn-glycero-3-phosphocholine (PGPC) is another major component of oxidized phospholipids found in atherosclerotic plaque (6). However, it remains unclear whether PGPC impairs endothelial function and contributes to the development of atherosclerosis.

Ferroptosis is a novel cell death mechanism mainly mediated by iron-dependent lipid peroxidation (7, 8, 9). Previous studies have found that the inactivation of glutathione peroxidase 4 (GPX4), an antioxidant enzyme that neutralizes lipid peroxides, can cause the accumulation of lipid reactive oxygen species (ROS) and eventually occurrence of ferroptosis (10). It has been shown that ferroptosis involved in the development of various cardiovascular diseases including cardiomyopathy, myocardial infarction, heart failure, myocardial ischemia-reperfusion injury, and vascular calcification (11, 12, 13, 14, 15, 16). Importantly, ferroptosis is involved in the development of atherosclerosis (17, 18, 19, 20, 21, 22). A previous study showed that induction of endothelial cell (EC) ferroptosis promotes atherosclerosis in Apolipoprotein E^−/−^ (*ApoE*^*−/−*^) mice (23). They found that oxidized low-density lipoprotein (oxLDL) can cause ferroptosis to damage ECs, suggesting that EC ferroptosis may contribute to atherosclerosis (23). Indeed, inhibition of ferroptosis alleviates atherosclerosis by reducing lipid peroxidation and endothelial dysfunction (23, 24). A previous study also showed that overexpressed GPX4 can improve endothelial function and inhibited atherosclerosis (25). More importantly, recent studies have shown that oxidized phospholipids can induce ferroptosis (26, 27, 28). It is possible that vascular ECs may uptake the oxidized phospholipids to induce ferroptosis contributing to endothelial dysfunction, which may be involved in the development of atherosclerosis. However, how ECs uptake oxidized phospholipids to induce endothelial dysfunction by promoting ferroptosis remains unclear. Whether PGPC (one of the major components of oxidized phospholipids) induces endothelial cell ferroptosis is unknown.

In the present study, we found that PGPC could impair endothelial function by inducing EC ferroptosis through the receptor of CD36 to increase fatty acid binding protein-3 (FABP3). E06, a natural antibody against oxidized palmitoylarachidonyl phosphatidylcholine IgM (2, 29), inhibits PGPC-induced EC ferroptosis. Our findings highlight the importance of some oxidized phospholipids in the promotion of atherosclerosis by inducing EC ferroptosis.

## Materials and methods

### Cell culture

Human umbilical vein endothelial cells (HUVECs) were purchased from ScienCell. HUVECs between passages 4 and 6 were used in all experiments. The HUVECs were plated in 6-well or 24-well or 96-well plates, and grown in endothelial cell medium (ECM, catalog no.: 1,001; ScienCell) containing 5% fetal bovine serum (FBS), 1% endothelial cell growth factor supplement, and 1% antibiotic and maintained at 37°C in a humidified atmosphere of 95% air and 5% CO_2_ until confluent. The cells were maintained in 0.5% FBS overnight before treatment (30).

### Cell viability assay

The Cell Counting Kit-(CCK-8) assay was used to determine whether PGPC causes cytotoxicity in HUVECs. Briefly, HUVECs (2 × 10^4^ cells/well) were plated in 96-well plates, and PGPC (catalog no.: 870602P; Avanti Polar Lipids, Inc) was then added to each well and incubated at 37°C. The cell viability was detected after incubation with 12.5, 25, or 50 μM PGPC for 12, 24, and 48 h, respectively, to observe the dose- and time-dependent effects. CCK-8 solution (10 μl, CellorLab, guangzhou, China) was then added to each well and incubated for 4 h at 37°C. The absorbance was measured at 450 nm using a microplate reader (Thermo). The percentage of living cells in the treated cultures was calculated relative to that in the untreated cultures.

### Quantitative real-time RT-PCR

The expression of various genes was analyzed using quantitative RT-PCR (qRT-PCR). Total RNA was extracted using TRIzol reagent (Sigma-Aldrich), according to the manufacturer's instructions. cDNA was synthesized using the Transcriptor First Strand cDNA Synthesis Kit (Roche). RT-PCR was carried out using a Light-Cycler®480 SYBR Green I Master (Roche). Primer sequences used were as follows: *FABP3*, forward: 5′-TATGGTGGACGCTTTCCTGG-3 and reverse: 5′-AACCCACACCGAGTGACTTC-3′; *FABP4*, forward: 5′-TAGATGGGGGTGTCCTGGTA-3′ and reverse: 5′-TCGTGGAAGTGACGCCTTTC-3′; *FABP5*, forward: 5′-GGAGCTAGGAGTGGGAATAGC-3′ and reverse: 5′-CTGATGCTGAACCAATGCACC-3′; *ACSL3*, forward: 5′-ACGTTCGTCCCCTCGCAT-3′ and reverse: 5′-GTTGGACGGGGTCGCATAC-3; *ACSL4*, forward: 5′-ACACTCTCTGACCAGTCCAGC-3′ and reverse: 5′-GCAGCCATAAGTGTGGGCTT-3; *CD36*, forward: 5′-GCAACAAACCACACACTGGG-3 and reverse: 5′-AGTCCTACACTGCAGTCCTCA-3; *GAPDH*, forward: 5′-AATGGGCAGCCGTTAGGAAA-3′ and reverse: 5′-GCGCCCAATACGACCAAATC-3′. All samples were analyzed using a real-time Bio-Rad analyzer.

### RNA interference (RNAi)

The gene expression was knocked down by RNA silencing using Lipofectamine® RNAiMAX and the corresponding manufacturer's protocol. siRNA (10 nmol each) was transfected into cells using Lipofectamine® RNAiMAX transfection reagent (catalog no.:13778150; Invitrogen) following the manufacturer's guidelines. The siRNA was synthesized by Guangzhou RiboBio Co., Ltd.

### Measurement of superoxide anion (O_2_^•−^) generation

The HUVECs were cultured to 90% confluence then serum starved with 0.5% FBS. Based on the cell viability data showing that 25 μM of PGPC and treatment for 24 h obtained significant effect, the HUVECs were pretreated with PGPC (25 μM), tumor necrosis factor alpha (TNF-α;10 μM; catalog no.: H8916; Sigma-Aldrich, as a positive control), and *N*-acetylcysteine (NAC; 1 mM; catalog no.: A7250; Sigma-Aldrich) for 24 h. Then, the cells were washed twice with Hank's balanced salt solution (HBSS) and incubated with dihydroethidium (DHE; 10 μM; catalog no.: D7008; Sigma-Aldrich) containing L-arginine (25 μM) and A23187 (5 μM) for 30 min at 37°C. Fluorescence images were obtained using a fluorescence microscope (DMi8; Leica, Wetzlar, Germany), and relative changes were analyzed using ImageJ software as previously described (31, 32).

### Immunofluorescence staining and immunohistochemistry

HUVECs were cultured to 90% confluence, serum-starved with 0.5% FBS, and treated with PGPC (25 μM) for 24 h. They were fixed in 4% paraformaldehyde at about 25°C for 30 min and then washed with phosphate buffer saline (PBS). Nonspecific immunoreactions were blocked using 5% bovine serum albumin (BSA) + 0.1% Triton X + 0.1% Tween 20 in PBS for 1 h at about 25°C. Cells were then washed in PBS and incubated with a primary antibody against FABP3 (1:200 dilution; catalog no.:10676-1-AP; Proteintech) overnight at 4°C. Following washing, the cells were incubated in goat anti-rabbit IgG secondary antibody conjugated to Alexa Fluor 488 (1:1,000 dilution; catalog no.:4412S; Cell Signaling Technology) overnight at 4°C. F-actin was stained with phalloidin (1:1,000 dilution; 8953S; Cell Signaling Technology). Cells were then washed with PBS and counterstained with Hoechst 33342 (1 μg/ml; catalog no.:4082S; Cell Signaling Technology) for 5 min. Images were obtained using a laser-scanning confocal microscope (LSM780; Carl Zeiss, Jena, Germany), and the relative changes were analyzed using ImageJ software. The atherosclerotic lesion tissues were collected at the First Affiliated Hospital, Sun Yat-sen University. Atherosclerotic lesion tissues were formalin-fixed and paraffin-embedded. This study was approved by the Ethics Review Board of the First Affiliated Hospital, Sun Yat-sen University, and abided by the Declaration of Helsinki principles. Written informed consent forms were obtained from all patients. Slides were dewaxed in xylene, boiled for 20 min in citrate buffer (10 mM, pH 6.0) for antigen retrieval, and then rehydrated for use with paraffin sections. Tissue slices were washed 3 times with PBS and blocked using a blocking solution (5% bovine serum albumin (BSA) + 0.1% Triton X + 0.1% Tween 20 in PBS). Sections were incubated with primary antibodies CD31 (1:1,000 dilution; catalog no.: 3528S; Cell Signaling Technology); CD31 (1:100 dilution; catalog no.: ab28364; Abcam) and GPX4 (1:500; catalog no.: ab125066; Abcam) overnight at 4°C. Sections were washed three times with PBS and incubated with appropriate Alexa Fluor 488 (1:1,000 dilution; catalog no.: 4412S; Cell Signaling Technology), Alexa Fluor 555 (1:1,000 dilution; catalog no.: 4413S; Cell Signaling Technology) conjugated secondary antibodies and horseradish peroxidase-coupled secondary antibody diluted 1:1,000 in blocking solution for 1 h at room temperature; washed again three times, and mounted on slides with Mounting medium with DAPI (catalog no.: ab104139; Abcam) or hematoxylin (catalog no.: G1080; Solarbio). Images were obtained using a laser-scanning confocal microscope (LSM780; Carl Zeiss, Jena, Germany) and a positive fluorescence microscope (BX63; Olympus, Japan) as previously described (5, 33).

### Measurement of mitochondrial reactive oxygen species (MtROS)

The intensity of MtROS was detected using confocal microscopy. MtSOX Deep Red (10 μM; catalog no.: MT14; DOJINDO) was used to incubate HUVECs to conduct fluorescence microscopy assay. The cells were then washed with PBS and counterstained with Hoechst 33342 (1 μg/ml; catalog no.:4082S; Cell Signaling Technology) for 5 min. Images were observed using a laser-scanning confocal microscope (LSM780; Carl Zeiss), and the relative changes were analyzed using ImageJ software.

### Western blot analysis

HUVECs were cultured in ECM supplemented with 5% FBS, 1% growth factor, and 1% penicillin/streptomycin. The cells were treated with PGPC (25 μM) ± ferrostatin-1 (Fer-1;1 μM; catalog no.: SML0583; Sigma-Aldrich) or E06 mAb (E06; 10 μg/ml, catalog no.:330001S, Avanti Polar Lipids, Inc) or erastin (5 μM; catalog no.: B1524; APExBIO) for 24 h. The cells were then washed three times with PBS and lysed in RIPA lysis buffer (catalog no.: 9806S; Cell Signaling Technology). Proteins were separated using SDS-PAGE and transferred to PVDF membranes. The membranes were blocked with 5% BSA in TBS with 0.1% Tween®20 detergent for 2 h at about 25°C. The primary antibodies against FABP3 (1:1,000; catalog no.: 0676-1-AP; Proteintech), FABP4 (1:1,000; catalog no.: ab92501; Abcam), FABP5 (1:1,000; catalog no.: 39926T; Cell Signaling Technology), ACSL4 (1:1,000; catalog no.: sc-271800; Santa Cruz), GPX4 (1:1,000; catalog no.: ab125066; Abcam), CD36 (1:1,000; catalog no.: ab133625; Abcam), and GAPDH (1:1,000; catalog no.: 97166S; Cell Signaling Technology) were used for detecting the proteins by overnight incubation at 4°C. The membranes were washed three times with Tris Buffered saline (TBS) with 0.1% Tween®20 detergent and incubated with a horseradish peroxidase-coupled secondary antibody (HRP Goat anti-Mouse/Rabbit IgG; 1:10,000; catalog no.: SA00001-1/SA00001-2; Proteintech) for 1 h at about 25°C. Protein bands were detected using a chemiluminescence detection kit (Millipore, Billerica, MA, USA). The blots were quantified using the ImageJ software as previously described (32, 34).

### Measurement of glutathione

Briefly, after HUVECs were pretreated with PGPC, the cells were washed once with PBS and centrifuged for fine collection. After the supernatant was abandoned, the protein removal reagent S solution was added to the cell precipitation, and the samples were freeze-thawed twice with liquid nitrogen and a 37°C water bath. Cells were centrifuged at 10,000 *g* for 5 min after 5 min of standing at 4°C or ice bath. The supernatant was used to determine the total glutathione content. Total glutathione in the cell lysates was measured using a glutathione detection kit (catalog no.: S0052, Beyotime), according to the manufacturer’s instructions.

### Measurement of mitochondrial membrane potential (MMP)

MMP was measured using the JC-1 MitoMP Detection Kit. The cells were treated with PGPC (25 μM) and stained with JC-1 (2 μM; catalog no.: MT09; Dojindo; Japan) for 30 min at 37°C protected from light, washed twice with PBS, and counterstained with Hoechst 33342 (1 μg/ml; catalog no.:4082S; Cell Signaling Technology) for 5 min. Images were obtained using a laser-scanning confocal microscope (LSM780; Carl Zeiss, Jena, Germany), and the red/green fluorescence intensity ratio was analyzed using ImageJ analysis software. In the healthy mitochondria, JC-1 aggregates to form a polymer in the mitochondrial matrix, which emits intense red fluorescence. In unhealthy mitochondria, JC-1 monomers are presented in the cytoplasm due to the decline/loss of mitochondrial membrane potential, which generates green fluorescence. Therefore, changes in the ratio of red fluorescence/green fluorescence reflected the change in mitochondrial membrane potential.

### Fluorescence-activated cell sorting (FACS) assays

The HUVECs were cultured to 90% confluence. They were then serum starved with 0.5% FBS and pretreated with PGPC (25 μM) ± Fer-1 (1 μM) and erastin (5 μΜ, as a positive control) for 24 h. Then, the cells were washed twice with HBSS and incubated with BODIPY™ 581/591 C11 (C11-BODIPY, Lipid Peroxidation Sensor; 5 μM; catalog no.: D3861; Invitrogen) for 30 min at 37°C. The stained cells were then washed twice with HBSS. FACS was performed as previously described (35). C11-BODIPY was applied to detect lipid peroxides. All FACS experiments were performed on BD flow cytometry (BD Biosciences) and results were analyzed using the FlowJo software 10.6.2 (Treestar).

### Transmission electron microscopy (TEM) assays

For TEM assays, after fixation with 2.5% glutaraldehyde fixative at room temperature, HUVECs were scraped down and deposited into a centrifuge tube. The cells were centrifuged at 1,000 RPM for 5 min and fixed in the dark for 30 min. Subsequently, the cells were dehydrated at room temperature in a graded series of ethanol and acetone solutions. Ultrathin sections were obtained using an ultramicrotome (Leica Microsystems) and stained with a 2% uranium acetate-saturated alcohol solution. The cuprum grids were observed under an HT7800/HT7700 transmission electron microscope (Hitachi), and images were captured.

### Ferrous iron content assay

FerroOrange (1 μM; catalog no.: F374; DOJINDO) was used to measure intracellular ferrous iron (Fe^2+^) according to the manufacturer's protocol. HUVECs were treated with PGPC (25 μM) ± Fer-1 (1 μM) or erastin (5 μM) for the indicated amount of time and stained with a final concentration of 1 μM FerroOrange or for 30 min at 37°C. The cells were then washed with PBS and counterstained with Hoechst 33342 (1 μg/ml; catalog no.:4082S; Cell Signaling Technology) for 5 min. Images were obtained using a laser scanning confocal microscope (LSM780; Carl Zeiss). Relative changes were analyzed using the ImageJ software. The mean fluorescence intensity of each group was normalized to that of the control group.

The aorta tissues isolated from humans were homogenized in Saline and PBS and followed by centrifugation. The blood samples were collected and stood for 1 h and centrifuged, then the serum was collected for testing. The levels of iron in the blank (ddH_2_O), ferrous iron standard solution, and test tissue samples were examined by using a Ferrous Iron Colorimetric Assay Kit (catalog no.: E-BC-K773-M, Elabscience, China) according to the manufacturer's instructions. The reaction mix was incubated at room temperature for 15 min. The absorbance at 562 nm was measured by using a microplate reader (BioTek SynergyH1, BioTek). This study was approved by the Ethics Review Board of the First Affiliated Hospital, Sun Yat-sen University, and abided by the Declaration of Helsinki principles. Written informed consent forms were obtained from all patients.

### Vasodilation study

The experimental protocol was approved by the Animal Ethics Commission of the First Affiliated Hospital, Sun Yat-sen University. All animals were purchased from the Experimental Animal Center of Sun Yat-sen University. A vasodilation assay was performed as described in our previous studies (36, 37, 38). Briefly, the aortas were isolated from C57BL6 mice or *ApoE*^*−/−*^ mouse after anesthesia with pentobarbital. For atherosclerosis modeling, *ApoE*^*−/−*^ mice were fed a Western diet (Junke Biological Co, LTD) for a total of 4 weeks. The *ApoE*^*−/−*^ + Fer-1 group mice were intraperitoneally injected with 1 mg/kg of Fer-1 every day for 4 weeks (23). The mice in *ApoE*^*−/−*^ group were received 100 μl of PBS by intragastric gavage. After 4-weeks treatment, the mice were sacrificed, and the aortas were isolated for further data analysis. Four 3 mm wide aortic rings were obtained and transferred to Krebs-solution (pH 7.4,119 mM NaCl; 25 mM NaHCO_3_; 1.6 mM CaCl_2_; 4.7 mM KCl; 1.2 mM KH_2_PO_4_; 1.2 mM MgSO_4_·7H_2_O; and 11.1 mM D-glucose). The aortic rings were equilibrated for 1 h, and the solution was changed every 15 min. Aortic rings were pretreated with PGPC (80 μM) or without Fer-1 (10 μM) and HTS01037 (pan-specific FABP inhibitor, 200 μM, catalog no.: HY-101503, MCE) or erastin (50 μM) for 30 min. Subsequently, the aortic rings were pre-constricted with 10^−7^ mol/L 5-hydroxy tryptamine (5-HT, catalog no.:50679; Sigma-Aldrich). Endothelium-dependent vasodilation was detected with 10^−7^–10^−4^ mol/L acetylcholine (Ach, catalog no.: A6625; Sigma-Aldrich). As a control, the aortic rings were not pretreated as previously described (4).

### Perls’ blue staining

Briefly, the aorta tissue sections isolated from mice were first deparaffinized and rehydrated. Perls’ blue staining was performed as previously described (39). For Perls’ blue staining, the sections were stained with a 1:1 mixture of Prussian blue (catalog no.: G1424; Solarbio) staining solution A and solution B for 30 min, washed twice with distilled water, stained with Prussian blue staining solution C for 3 min, and washed with running water. They were dehydrated with three changes of 100% ethanol and two changes of xylene for 5 min each and sealed with neutral balsam. Images were obtained using a positive fluorescence microscope (BX63; Olympus).

### Statistical analysis

Statistical analyses were performed using GraphPad Prism 8.0 (GraphPad Software). Significant differences in mean values were determined using one-way analysis of variance (ANOVA), followed by Tukey’s test for more than two groups or Student's *t* test for two groups (*P* < 0.05). Data are expressed as mean ± SEM.

## Results

### PGPC caused ferroptosis in ECs

**Fig. 1:**
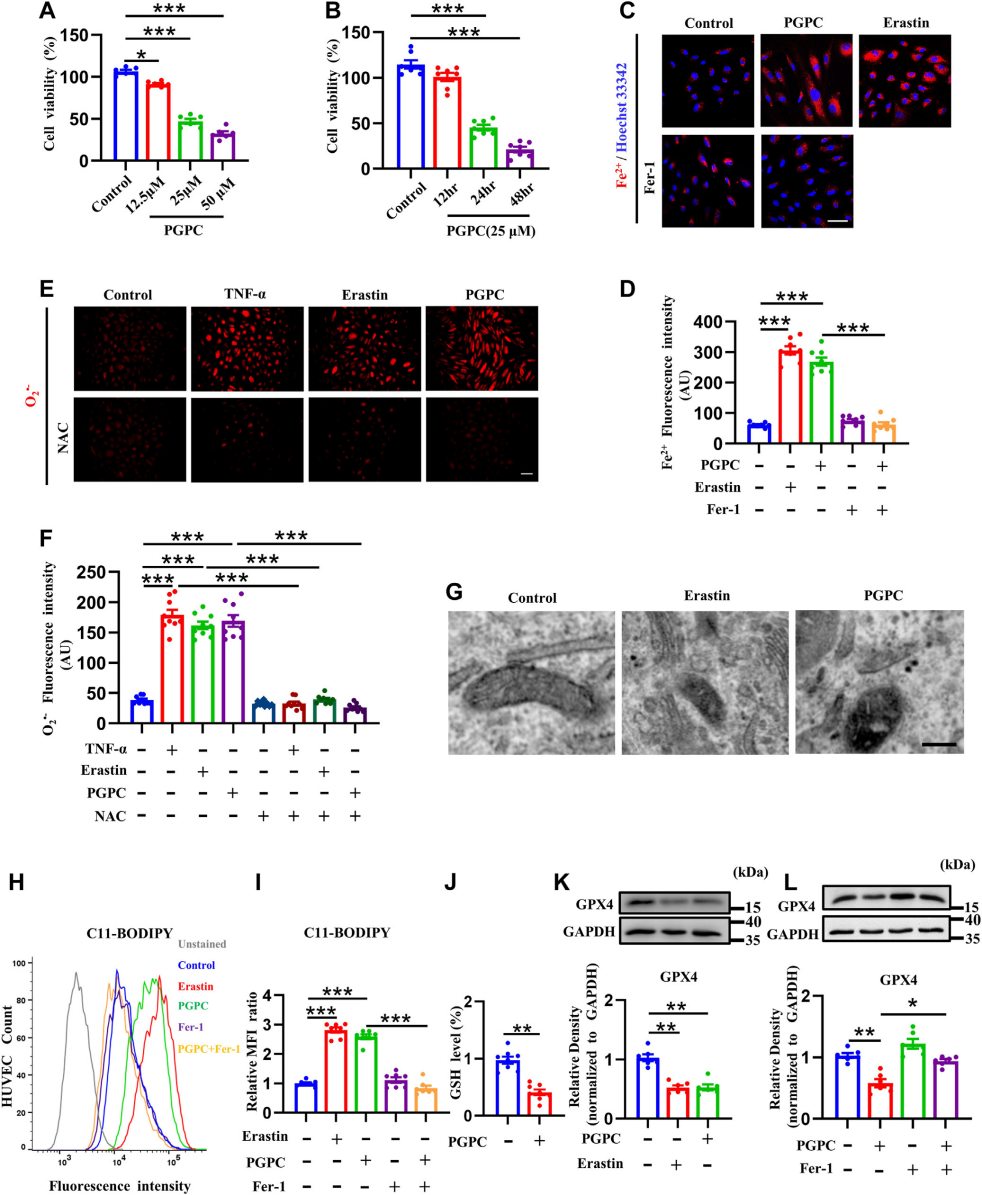
PGPC induced ferroptosis in human umbilical vein endothelial cells (HUVECs). A: CCK8 analysis of HUVECs treated with 12.5, 25, 50 μM PGPC for 24 h. (∗*P* < 0.05, ∗∗∗*P* < 0.001, n = 6). B: CCK8 analysis of HUVECs treated with 25 μM PGPC for 12, 24, 48 h. (∗∗∗*P* < 0.001, n = 7). C, D: Probe FerroOrange staining fluorescence (red) and bar chart showing the intracellular levels of ferrous iron (Fe^2+^) in cultured HUVECs after pretreatment with PGPC with or without Ferrostatin-1 (Fer-1) or erastin for 24 h. Erastin was used as a positive control. The nuclei were stained with Hoechst 33342 (blue). Scale bar represents 50 μm. (∗∗∗*P* < 0.001, n = 8). E, F: Dihydrothidium staining fluorescence (red) and a bar chart showing the intracellular levels of superoxide anion (O_2_^•−^) in cultured HUVECs after pretreatment with or without *N*-acetylcysteine (NAC), which were then exposed to tumor necrosis factor alpha (TNF-α), erastin, and PGPC. TNF-α was used as a positive control. The scale bar represents 100 μm. (∗∗∗*P* < 0.001, n = 9). G: Representative transmission electron microscopy (TEM) images of mitochondria in HUVECs after erastin and PGPC treatment for 24 h. Scale bar represents 2 μm. H: Representative fluorescence-activated cell sorting (FACS) data for C11-BODIPY labeling of HUVECs following PGPC treatment with or without Fer-1 or erastin for 24 h. Unstained C11-BODIPY was not added. HUVEC count indicates the number of HUVECs. I: Statistical analysis of mean fluorescence intensity (MFI) of C11-BODIPY. (∗∗∗*P* < 0.001, n = 7). J: Relative glutathione (GSH) levels in HUVECs after PGPC treatment for 24 h. (∗∗*P* < 0.01, n = 8). K, L: Western blots and bar chart showing the expression levels of glutathione peroxidase 4 (GPX4) in HUVECs treated with PGPC with or without Fer-1 or erastin for 24 h (∗*P* < 0.05, ∗∗*P* < 0.01, n = 6).

To determine whether PGPC can induce the death of vascular ECs, cell viability after treatment with 12.5, 25, 50 μM PGPC were detected. Figure 1A shows that PGPC can induce the death of HUVECs in a dose-dependent manner. In instance, 25 μM of PGPC treatment significantly reduced the cell viability of HUVECs (Fig. 1A). Accordingly, we decided to use 25 μM PGPC in our subsequent cell-cultured experiments. The effects of PGPC on HUVEC proliferation after 12, 24, and 48 h of treatment were also assessed using the CCK-8 assay. As seen in Fig. 1B, the cell viability was significantly altered after exposed to PGPC (25 μM) for 24 h with a time-dependent effect. To investigate whether PGPC causes ferroptosis in HUVECs, intracellular Fe^2+^ was measured in HUVECs treated with PGPC. Figure 1C, D showed that both erastin, an activator of ferroptosis, and PGPC increased Fe^2+^ levels. PGPC-induced increase in Fe^2+^ levels was inhibited by Fer-1, an inhibitor of erastin-induced ferroptosis (Fig. 1C, D). Both PGPC and erastin significantly stimulated O_2_^•−^ production (Fig. 1E, F). NAC, an antioxidant, almost completely prevented PGPC-induced O_2_^•−^ generation (Fig. 1E, F). Ferroptosis is characterized by the formation of mitochondria that are smaller than usual and have condensed densities in the mitochondrial membrane (9, 40). Figure 1G shows that the mitochondria were smaller and had more condensed mitochondrial membranes in the PGPC group than in the control group. PGPC also increased the expression of the lipid peroxidation sensor C11-BODIPY, which was reduced by Fer-1 (41) (Fig. 1H, I). Furthermore, PGPC decreased GSH levels (Fig. 1J). Finally, we measured the level of GPX4, a protein associated with lipid peroxidation. Both PGPC and erastin reduced the expression of GPX4 (Fig. 1K), whereas Fer-1 reversed the PGPC-induced inhibition of GPX4 expression (Fig. 1L). These findings suggest that PGPC induces ferroptosis in HUVECs.

### PGPC activated FABP3 in ECs

**Fig. 2:**
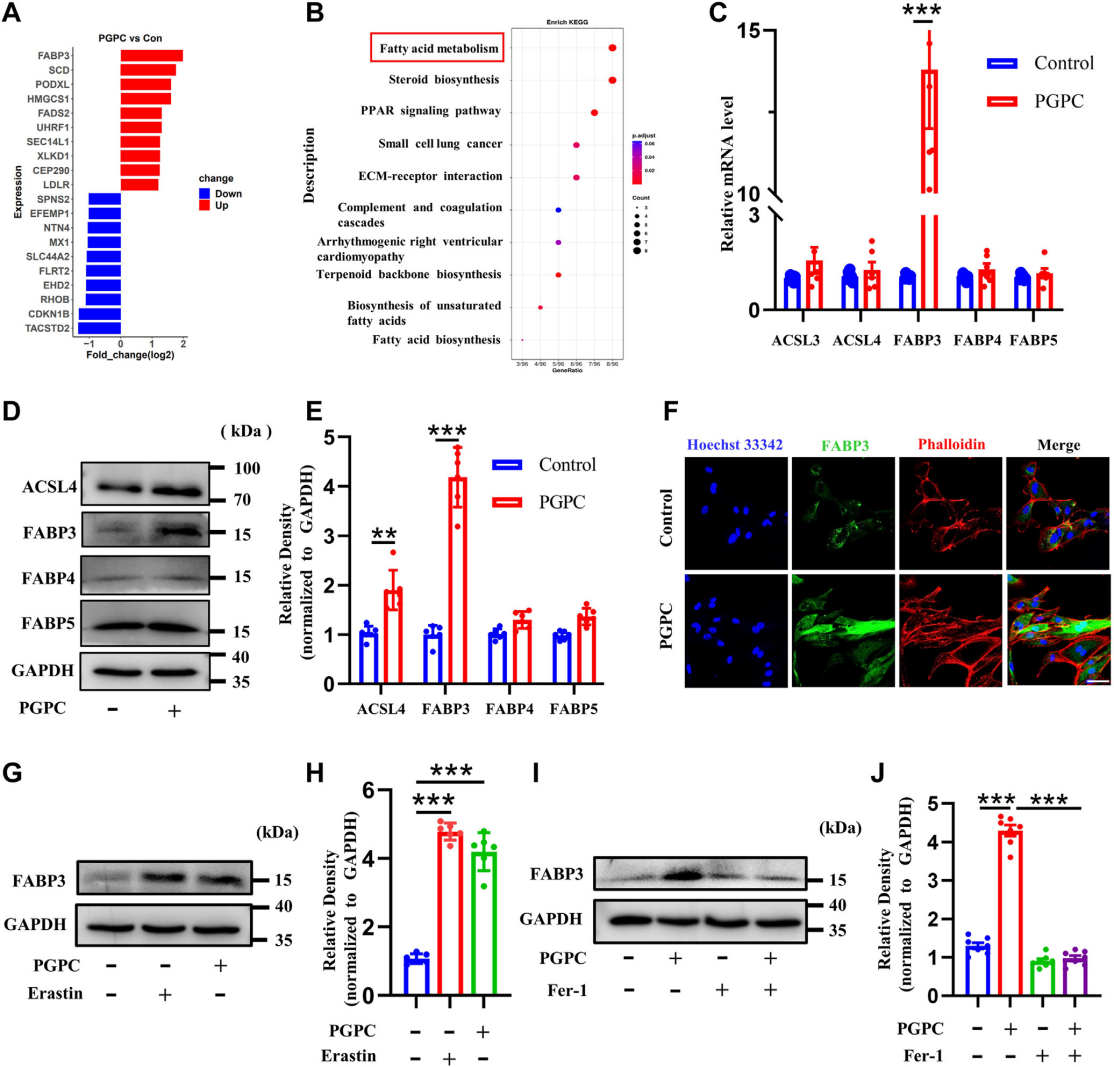
PGPC and ferroptosis promote fatty acid binding protein-3 (FABP3) expression in endothelial cells. A, B: Human umbilical vein endothelial cells (HUVECs) were pretreated with PGPC (25 μM) media for 24 h followed by mass spectrometry and enriched Kyoto Encyclopedia of Genes and Genomes analysis for regulated proteins. C: qRT-PCR showing the intracellular mRNA levels of *ACSL3*, *ACSL4*, *FABP3*, *FABP4*, and *FABP5* in HUVECs after pretreatment with PGPC for 24 h (∗∗∗*P* < 0.001, n = 6). D, E: Western blots and bar charts showing the protein levels of ACSL4, FABP3, FABP4, and FABP5 in HUVECs after pretreatment with PGPC for 24 h (∗∗*P* < 0.01, ∗∗∗*P* < 0.001, n = 6). F: Immunofluorescence microscopy showing an increase in fluorescence intensity of FABP3 (green) after treatment of cultured HUVECs with PGPC for 24 h. F-actin was stained with phalloidin (red). The nuclei were stained with Hoechst 33342 (blue). Scale bar represents 50 μm. G, H: Western blots and bar chart showing FABP3 expression levels in HUVECs treated with PGPC or erastin for 24 h (∗∗∗*P* < 0.001, n = 6). I, J: Western blots and bar chart showing FABP3 expression levels in HUVECs treated with PGPC with or without Fer-1 for 24 h (∗∗∗*P* < 0.001, n = 7).

To further explore the mechanisms by which PGPC induced ferroptosis, we performed a comparative proteome study in HUVECs treated with or without PGPC. The Enriched Kyoto Encyclopedia of Genes and Genomes revealed a distinct difference between the control and PGPC treatment. Among these differences, the expression of FABP3 and fatty acid metabolism signaling pathways showed the most striking changes (Fig. 2A, B). Next, the fatty acid metabolic pathway genes *ACSL3*, *ACSL4*, *FABP3*, *FABP4*, and *FABP5* were examined in PGPC-treated endothelial cells (ECs), with *FABP3* expression being the most pronounced (Fig. 2C). Western blotting also showed that PGPC significantly upregulated the expression of FABP3 (Fig. 2D, E), and immunofluorescence staining showed that FABP3 was mainly expressed in the cytoplasm (Fig. 2F). Both the ferroptosis activators (erastin and PGPC) increased the expression of FABP3 (Fig. 2G, H), and the ferroptosis inhibitor Fer-1 significantly reduced the upregulation of FABP3 induced by PGPC (Fig. 2I, J). These dates clearly show that FABP3 plays a significant regulatory role in PGPC-induced ferroptosis.

### Knockdown of FABP3 rescued PGPC-induced ferroptosis

**Fig. 3:**
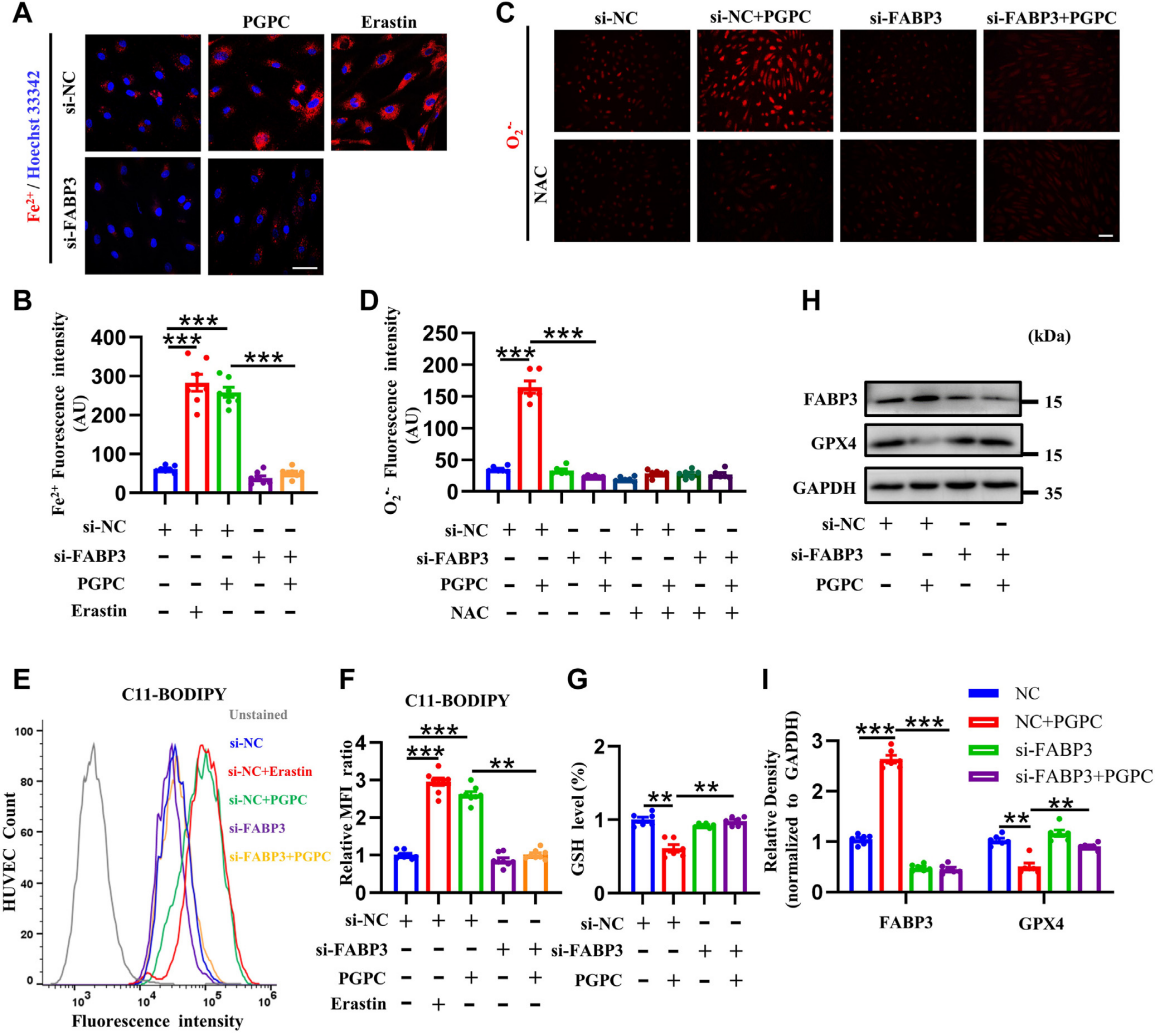
PGPC induces ferroptosis via upregulating fatty acid binding protein-3 (FABP3) in endothelial cells. A, B: Probe FerroOrange staining fluorescence (red) and bar chart showing the intracellular levels of ferrous iron (Fe^2+^) in cultured human umbilical vein endothelial cells (HUVECs) after knockdown of FABP3 followed with PGPC treatment with or without erastin for 24 h. Nuclei were stained with Hoechst 33342 (blue). Scale bar represents 50 μm. (∗∗∗*P* < 0.001, n = 7). C, D: Dihydrothidium staining fluorescence (red) and bar chart showing the intracellular levels of superoxide anions (O_2_^•−^) in cultured HUVECs after pretreatment with or without *N-*acetylcysteine (NAC), which were then exposed to PGPC after knockdown of FABP3. Scale bar represents 100 μm. (∗∗∗*P* < 0.001, n = 6). E: The levels of C11-BODIPY in negative control and FABP3-knockdown HUVECs following PGPC treatment for 24 h were determined using fluorescence-activated cell sorting (FACS). HUVEC count indicates the number of HUVECs. F: Mean fluorescence intensity (MFI) values of C11-BODIPY in each group. (∗∗*P* < 0.01, ∗∗∗*P* < 0.001, n = 8). G: Relative glutathione (GSH) levels in negative control knockdown and FABP3-knockdown HUVECs following PGPC treatment for 24 h were determined. (∗∗*P* < 0.01, n = 6). H, I: Western blots and bar charts showing the levels of glutathione peroxidase 4 (GPX4) and FABP3 expression in negative control knockdown and FABP3-knockdown HUVECs after PGPC treatment for 24 h (∗∗*P* < 0.01, ∗∗∗*P* < 0.001, n = 6). si-FABP3, specific FABP3 siRNA; si-NC, negative control siRNA.

To verify whether PGPC induces ferroptosis by upregulating FABP3, FABP3 was knocked down in the HUVECs. *FABP3*-siRNA2 and *FABP3*-siRNA3 were selected to knock down FABP3 because of their maximal silencing effects (supplemental Fig. S1A). When FABP3 was knocked down, the amount of intercellular Fe^2+^ induced by PGPC was significantly reduced (Fig. 3A, B). Similarly, *FABP3* silencing remarkably decreased PGPC-induced O_2_^•−^ generation (Fig. 3C, D). Additionally, FABP3 knockdown significantly reversed the PGPC-induced increase in the lipid peroxidation sensor C11-BODIPY (Fig. 3E, F) and the PGPC-induced reduction in glutathione (Fig. 3G). FABP3 knockdown also reversed the degradation of GPX4 protein induced by PGPC (Fig. 3H, I). Collectively, our findings indicate that FABP3 knockdown prevents PGPC-induced ferroptosis.

### Mitochondrial damage and dysfunction involved in PGPC-induced ferroptosis via FABP3

**Fig. 4:**
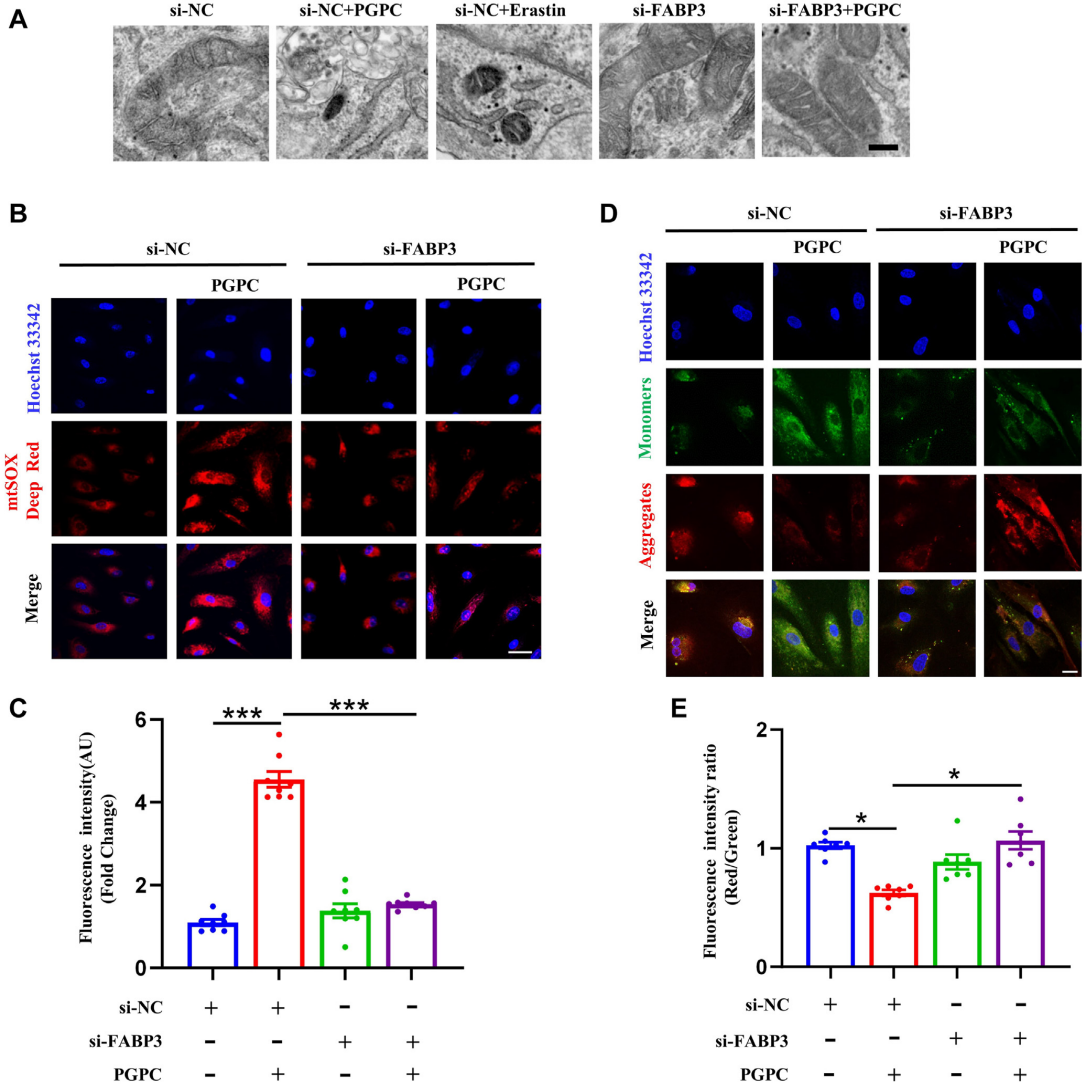
Mitochondrial reactive oxygen species (MtROS) were involved in ferroptosis and mitochondrial dysfunction induced by PGPC via fatty acid binding protein-3 (FABP3) in endothelial cells. A: Representative transmission electron microscopy (TEM) images of mitochondria in negative control knockdown, FABP3-knockdown human umbilical vein endothelial cells (HUVECs) following PGPC treatment for 24 h. Scale bar represents 2 μm. B, C: MitoSOX Red staining fluorescence (red) and bar chart showing the intracellular levels of mitochondrial ROS in negative control knockdown, FABP3-knockdown following PGPC treatment for 24 h in cultured HUVECs. The nuclei were stained with Hoechst 33342 (blue). The scale bar represents 40 μm. (∗∗∗*P* < 0.001, n = 8). D, E: JC-1 staining fluorescence (red and green) and bar chart showing mitochondrial membrane potential (MMP) in negative control knockdown, FABP3-knockdown HUVECs following PGPC treatment for 24 h after pretreatment of cultured HUVECs with PGPC. Red represents aggregated JC-1 in the mitochondrial matrix, green represents JC-1 monomers in the cytoplasm of mitochondria. The ratio of red fluorescence to green fluorescence of the control was defined as 1. The nuclei were stained with Hoechst 33342 (blue). Scale bar represents 20 μm. (∗*P* < 0.05, n = 7). si-FABP3, specific FABP3 siRNA; si-NC, negative control siRNA.

TEM images revealed that FABP3 knockdown reversed PGPC-induced mitochondrial damage, allowing further investigation of the role of mitochondria in PGPC-induced ferroptosis (Fig. 4A). Additionally, PGPC augmented the levels of MtROS, and FABP3 knockdown reversed the increase in MtROS levels induced by PGPC (Fig. 4B, C). The intensity of green fluorescence significantly increased after PGPC treatment but recovered to normal after FABP3 knockdown, indicating that PGPC impairs mitochondrial function by reducing MMP through FAPB3 (Fig. 4D, E). These results demonstrate that PGPC impairs mitochondrial function via FABP3, triggering ferroptosis in ECs.

### CD36 modulated PGPC-induced ferroptosis by regulating FABP3 levels

**Fig. 5:**
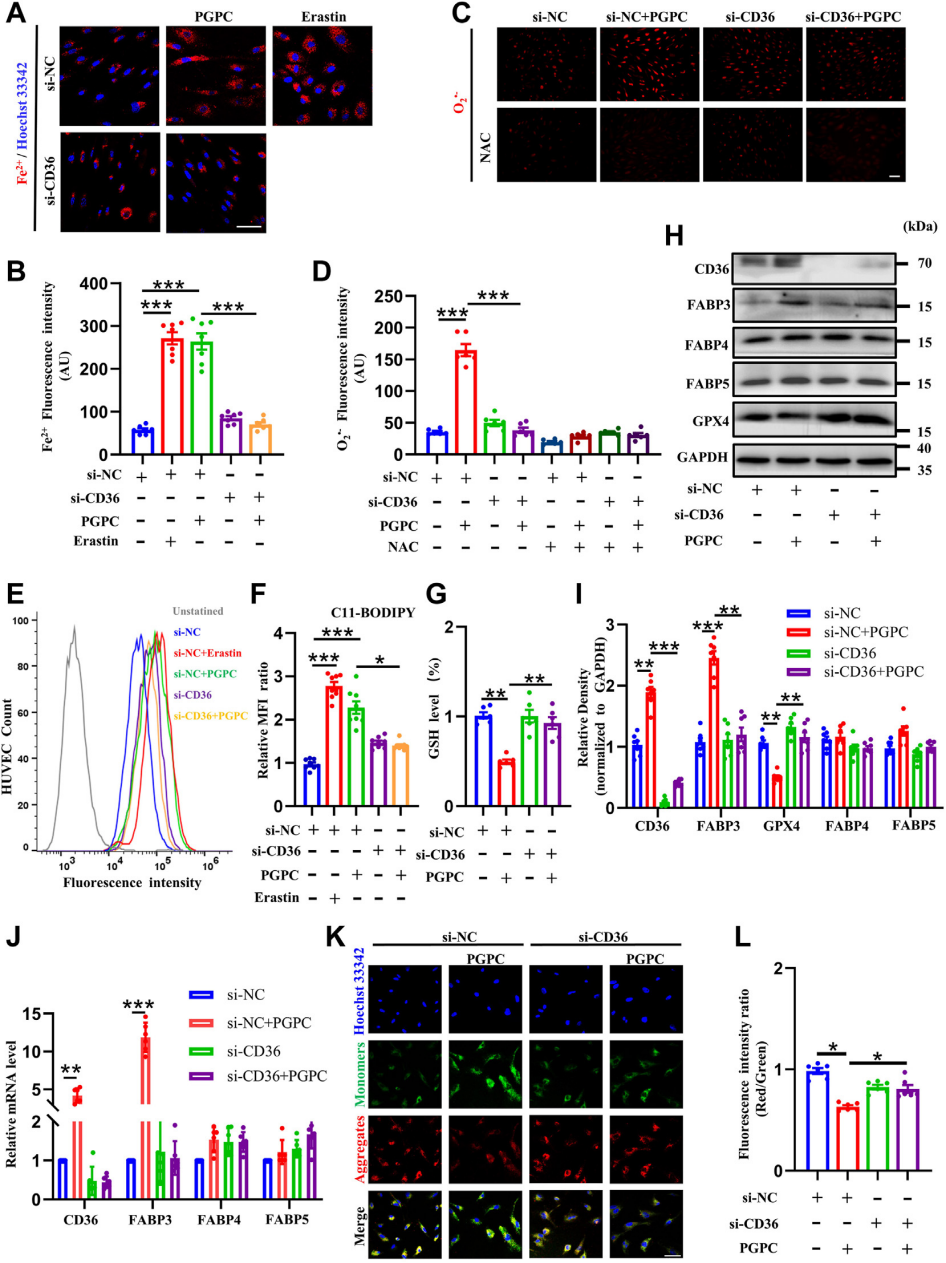
CD36 participates in PGPC-induced ferroptosis through its interaction with PGPC in endothelial cells. A, B: Probe FerroOrange staining fluorescence (red) and bar chart showing the levels of ferrous iron (Fe^2+^) in negative control knockdown, CD36-knockdown following PGPC treatment for 24 h in cultured human umbilical vein endothelial cells (HUVECs). Nuclei were stained with Hoechst 33342 (blue). Scale bar represents 50 μm. (∗∗∗*P* < 0.001, n = 7). C, D: Dihydroethidium staining fluorescence (red) and bar chart showing the intracellular levels of superoxide anion (O_2_^•−^) in negative control and CD36-knockdown HUVECs after pretreatment of cultured endothelial cells with PGPC and *N-*acetylcysteine (NAC). The scale bar represents 100 μm. (∗∗∗*P* < 0.001, n = 6). E, F: C11 BODIPY staining using followed fluorescence-activated cell sorting (FACS) analysis and bar chart showing the lipid peroxidation in negative control knockdown, CD36-knockdown HUVECs following PGPC treatment for 24 h. HUVEC count indicates the number of HUVECs. (∗*P* < 0.05, ∗∗∗*P* < 0.001, n = 8). G: Relative glutathione (GSH) levels in negative control knockdown, CD36-knockdown HUVECs following PGPC treatment for 24 h. (∗∗*P* < 0.01, n = 6). H, I: Western blots and bar charts showing the levels of CD36, FABP3, FABP4, FABP5, and glutathione peroxidase 4 (GPX4) expression in negative control and CD36-knockdown HUVECs following PGPC treatment for 24 h. (∗∗*P* < 0.01, ∗∗∗*P* < 0.001, n = 7). J: qRT-PCR showing the intracellular mRNA levels of *FABP3*, *FABP4*, *FABP5* in HUVECs after in negative control and CD36-knockdown HUVECs following PGPC treatment for 24 h. (∗∗*P* < 0.01, ∗∗∗*P* < 0.001, n = 6). K, L: JC-1 staining fluorescence (red and green) and bar chart showing mitochondrial membrane potential (MMP) of PGPC-treated HUVECs for 24 h after negative control knockdown and CD36-knockdown in HUVECs. Red represents aggregated JC-1 in the mitochondrial matrix, green represents JC-1 monomers in the cytoplasm of mitochondria. The ratio of red fluorescence to green fluorescence of the control was defined as 1. Nuclei were stained with Hoechst 33342 (blue). Scale bar represents 50 μm. (∗*P* < 0.05, n = 6). si-*CD36*, specific *CD36* siRNA; si-NC, negative control siRNA.

Numerous studies have shown that CD36 participates in the development of atherosclerosis and that genetic deletion of CD36 or blocking of the CD36-induced signaling cascade decreases the formation of atherosclerotic lesions. Oxidized phospholipids, oxidized low-density lipoproteins, and long-chain fatty acids are a few of the many ligands to which CD36 may bind. Because PGPC is an oxidized phospholipid, it is possible that PGPC increases FABP3 expression in HUVECs through interactions with CD36. Therefore, CD36 was silenced in HUVECs and *CD36*-siRNA2 was selected for CD36 knockdown because of its maximum silencing effect (supplemental Fig. S1B). CD36 knockdown significantly decreased PGPC-induced Fe^2+^ overload (Fig. 5A, B) and O_2_^•−^generation (Fig. 5C, D). Furthermore, CD36 knockdown significantly decreased the intensity of the lipid peroxidation sensor C11-BODIPY induced by PGPC (Fig. 5E, F) and reversed the PGPC-induced degradation of glutathione (Fig. 5G). Western blotting confirmed that CD36 knockdown reversed PGPC-induced degradation of GPX4 and upregulation of FABP3 (Fig. 5H, I). We found that expression of FABP4 and FABP5 did not change by PGPC after silencing CD36. Additionally, qRT-PCR demonstrated that PGPC promoted the transcriptional expression of FABP3 via CD36 (Fig. 5J). The intensity of green fluorescence recovered to normal after CD36 knockdown in HUVECs following PGPC treatment, indicating that PGPC reduced MMP via the CD36 receptor (Fig. 5K, L). Taken together, these dates suggest that PGPC causes EC ferroptosis by upregulating FABP3 via the receptor CD36.

### E06 antibody reversed PGPC-induced ferroptosis

**Fig. 6:**
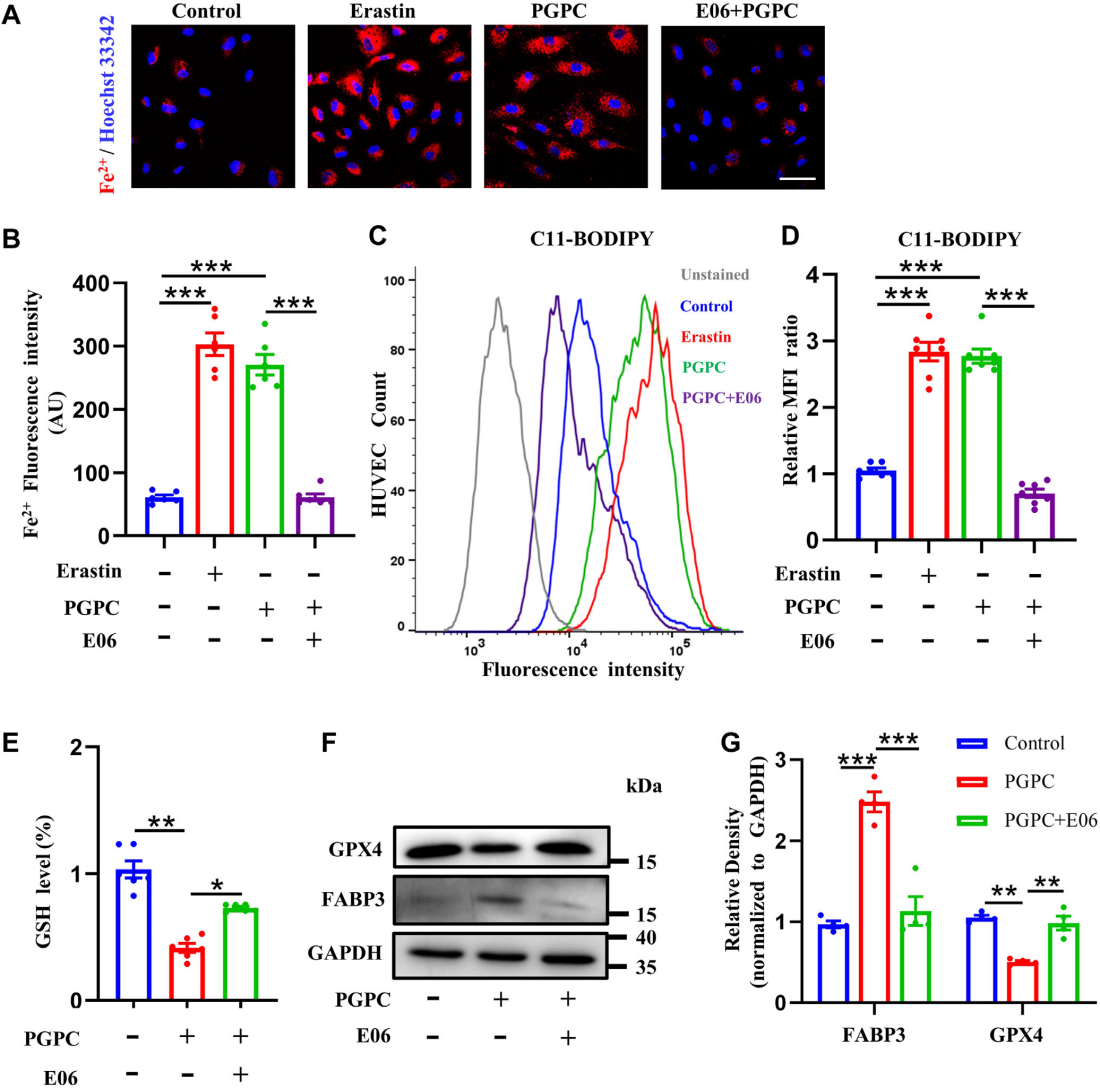
E06 rescues ferroptosis induced by PGPC in endothelial cells. A, B: Probe FerroOrange staining fluorescence (red) and bar chart showing the intracellular levels of ferrous iron (Fe^2+^) after pretreatment of cultured human umbilical vein endothelial cells (HUVECs) with erastin and PGPC with or without E06 (10 μg/ml) for 24 h. Nuclei were stained with Hoechst 33342 (blue). Scale bar represents 50 μm. (∗∗∗*P* < 0.001, n = 6). C, D: C11 BODIPY staining using fluorescence-activated cell sorting (FACS) analysis and bar chart showing lipid peroxidation in HUVECs following erastin and PGPC with or without E06 treatment for 24 h. HUVEC count indicates the number of HUVECs. (∗∗∗*P* < 0.001, n = 7). E: Relative glutathione (GSH) levels in HUVECs after PGPC treatment with or without E06 for 24 h were determined. (∗*P* < 0.05, ∗∗*P* < 0.01, n = 6). F, G: Western blots and bar charts showing the protein levels of fatty acid binding protein-3 (FABP3) and glutathione peroxidase 4 (GPX4) after pretreatment of cultured HUVECs with PGPC with or without E06 treatment for 24 h. (∗∗*P* < 0.01, ∗∗∗*P* < 0.001, n = 4).

The natural IgM antibody E06 can bind to the phosphocholine head group of oxidized phospholipids, limit the absorption of oxidized low-density lipoprotein by macrophages, and suppress the proinflammatory characteristics of oxidized phospholipids. We further determined whether E06 inhibited PGPC-induced ferroptosis in HUVECs. E06 significantly decreased the Fe^2+^ overload (Fig. 6A, B) and lipid peroxidation induced by PGPC (Fig. 6C, D). Similarly, E06 reversed PGPC-induced degradation of glutathione (Fig. 6E). Moreover, Western blotting confirmed that the E06 antibody reversed PGPC-induced degradation of GPX4 and upregulation of FABP3 (Fig. 6F, G). These findings suggest that the E06 antibody can reverse PGPG-induced ferroptosis.

### PGPC inhibited endothelium-dependent vasodilation by inducing ferroptosis

**Fig. 7:**
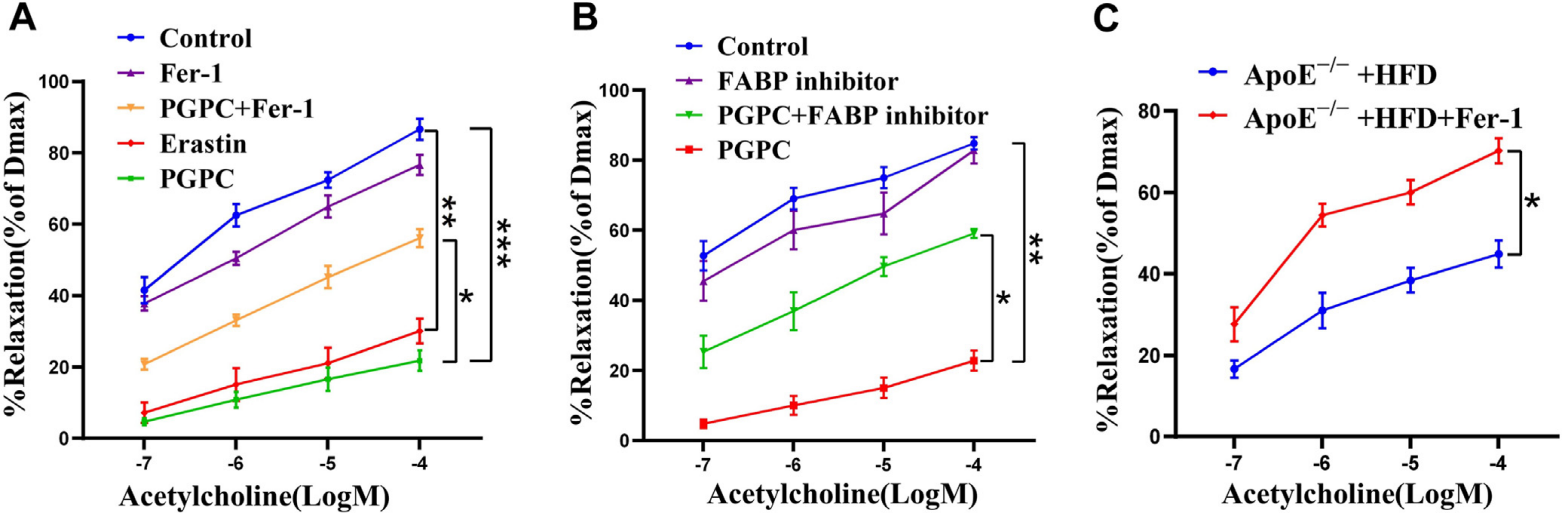
PGPC impaired endothelium-dependent vasodilation by fatty acid binding protein-3 (FABP3)-mediated ferroptosis. A, B: Line chart showing the endothelium-dependent vasodilation of aortic rings ex vivo. Aortic rings isolated from C57BL6 were pretreated with PGPC or without Ferrostatin-1 (Fer-1), erastin, HTS01037 for 30 min. Subsequently, aortic rings were pre-constricted with 5-hydroxy tryptamine (5-HT). Endothelium-dependent vasodilation was detected using *N-*acetylcholine (Ach). (∗*P* < 0.05, ∗∗*P* < 0.01, ∗∗∗*P* < 0.001, n = 6). C: Line chart showing the endothelium-dependent vasodilation of aortic rings ex vivo. Aortic rings isolated from high fed diet Apolipoprotein E^−/−^ mice intraperitoneally injected with or without 1 mg/kg of Fer-1 every day for 4 weeks. Subsequently, aortic rings were pre-constricted with 5-hydroxy tryptamine (5-HT). Endothelium-dependent vasodilation was detected using acetylcholine (Ach). (∗*P* < 0.05, n = 7).

Finally, we examined the effects of PGPC on endothelium-dependent vasodilation. Both PGPC and erastin significantly inhibited endothelium-dependent vasodilation compared to the control group. PGPC-induced impairment of endothelium-dependent vasodilation was partially inhibited by Fer-1 (Fig. 7A), indicating that PGPC-impaired endothelium-dependent vasodilation by inducing ferroptosis. Western blot analysis revealed that the FABP inhibitor HTS01037 downregulated FABP3 expression in HUVECs (supplemental Fig. S2). Furthermore, HTS01037 partially inhibited PGPC-impaired endothelium-dependent vasodilation (Fig. 7B). Collectively, the above results indicate that PGPC inhibits endothelium-dependent vasodilation, at least in part, by upregulating FABP3 expression. Moreover, we examined vascular reactivity in *ApoE*^*−/−*^ mice treated with Fer-1. Our findings demonstrated that, in contrast to high-fed diet *ApoE*^*−/−*^ mice, Fer-1 therapy clearly increased vascular reactivity (Fig. 7C). This finding provided additional evidence supporting the notion that ferrostatin-1 would attenuate atherosclerosis in *ApoE*^*−/−*^ mice by restoring endothelium-dependent vasodilation.

### Ferroptosis occurs in ECs of atherosclerotic lesions

**Fig. 8:**
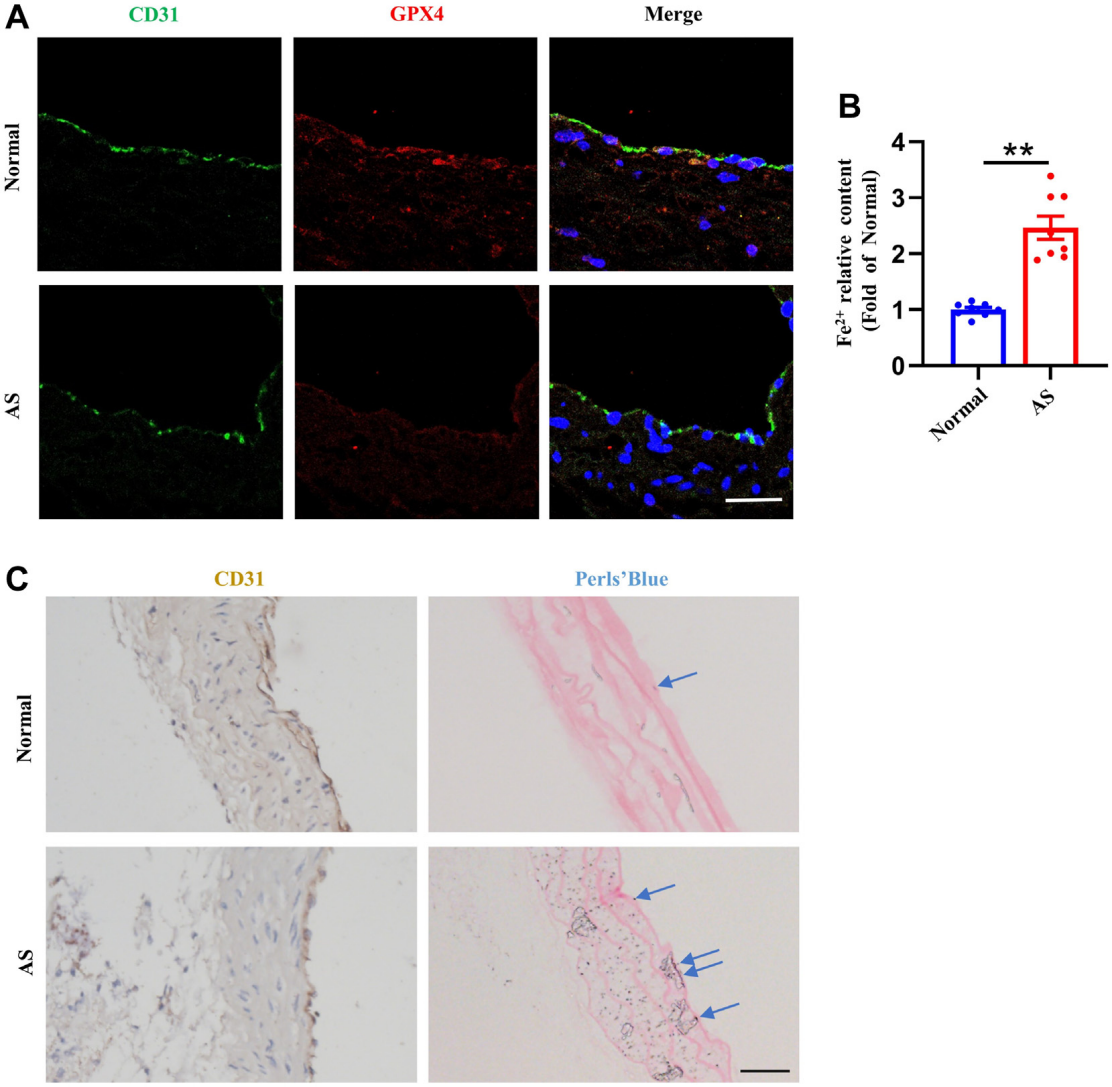
Ferroptosis occurs in endothelial cells of atherosclerotic vessels. A: Immunohistochemistry microscopy shows a decrease in the fluorescent intensity of CD31 (green) and in fluorescent intensity of GPX4 (red) in endothelial cells of atherosclerotic vessels compared with healthy individuals. Nuclei were stained with Hoechst 33342 (blue). Scale bar represents 50 μm. AS: atherosclerotic vessels tissues of patients. B: The ferrous iron levels in human aorta sections were measured by Ferrous Iron Colorimetric Assay Kit. (∗∗*P* < 0.01, n = 8). AS: atherosclerotic lesions tissues of patients. C: Representative images of mouse aorta sections stained with Perls Prussian blue, CD31 immunohistochemistry. Nuclei were stained with eosin (red) in Perls Prussian blue staining and hematoxylin (blue) in CD31 immunohistochemistry. Blue arrows indicate iron. Scale bar represents 40 μm. AS, atherosclerotic lesions tissues of the mouse.

To reveal if ferroptosis occurs in ECs of human atherosclerotic lesions, we measured aortic ferrous iron levels and ferroptosis markers by immunohistochemistry in paraffin-embedded tissue samples obtained from patients with atherosclerotic lesions. In comparison to histologically normal human arteries, atherosclerotic plaques exhibited a reduction of GPX4 levels in ECs (Fig. 8A). Ferrous iron content assay revealed that aortic tissues from patients with atherosclerotic lesions had higher levels of ferrous iron deposits than in non-atherosclerotic aortic tissues (Fig. 8B). In addition, Perls’ blue staining revealed that ECs from high-fed diet *ApoE*^*−/−*^ mice with atherosclerotic lesions had higher levels of iron deposits than in non-atherosclerotic tissue samples (Fig. 8C). All of these findings suggested that ECs in atherosclerotic lesions undergo ferroptosis.

## Discussion

Endothelial dysfunction is the early stage of atherosclerosis. We found that PGPC impairs endothelial function by inducing ferroptosis, which may contribute to the development of atherosclerosis. The present study had three novel findings: *1*) PGPC impairs endothelial function by inducing EC ferroptosis; *2*) PGPC induces EC ferroptosis through the CD36 receptor to increase FABP3 expression; *3*) E06 inhibited PGPC-induced EC ferroptosis.

PGPC is a major component of oxidized phospholipids in atherosclerotic plaques (6). In the present study, we found that PGPC inhibits endothelium-dependent vasodilation. Since endothelial dysfunction is an early stage of atherosclerosis (42, 43, 44), our findings suggest that PGPC may participate in the development of atherosclerosis. We further found that PGPC induced EC ferroptosis and inhibited ferroptosis, partially restoring PGPC-impaired endothelium-dependent vasodilation. Previous studies have shown that ferroptosis is involved in the development of atherosclerosis (17, 18, 19, 20, 21, 22). The inhibition of EC ferroptosis alleviates atherosclerosis by improving endothelial function (23, 24). These studies, in agreement with our findings, suggested that PGPC induces atherosclerosis by inducing EC ferroptosis. A recent study reported that the oxidized phosphatidylcholines POVPC and PONPC, which are also found in atherosclerotic plaques, can induce ferroptosis (28). Therefore, the components of oxidized phospholipids found in atherosclerotic plaques (including PGPC, POVPC, and PONPC) may induce atherosclerosis via EC ferroptosis.

Evidence for the importance of oxidized phospholipids in atherosclerosis comes from the accumulation of oxidized phospholipids in plaques (1). There was an EPIC Norfolk study that proved levels of oxidized phospholipids in plasma are associated with an increased risk of coronary artery disease (CAD) (45). The studies showed that the total phospholipid level in the plasma of patients is higher than that in the plasma of healthy individuals (46). Previous studies have shown that PGPC concentrations in plasma from healthy controls range from 0.15 to 1 μM (47, 48). In our preliminary study, we found that the plasma concentrations of PGPC increased significantly in patients with coronary artery disease compared to healthy individuals. A previous study demonstrated that PGPC (50 μM) can significantly induce apoptosis in vascular smooth muscle cells (48). We found that 25 μM of PGPC already significantly inhibited endothelial cell viability. Therefore, we chose 25 μM of PGPC to perform our experiments in cultured ECs in the present study.

FABP3 is widely expressed in the heart and brain and has been used as a novel marker for heart damage (49, 50). Previous studies suggest that FABP3 is associated with atherosclerosis (51, 52). FABP3 knockdown exerts anti-atherogenic effects by reducing the production of foamy macrophages (52). In the present study, we found that PGPC increased FABP3 expression in ECs. Inhibition of FABP3 restored PGPC-induced ferroptosis and PGPC-impaired endothelium-dependent vasodilation, demonstrating that PGPC induces ferroptosis by increasing FABP3 expression. Recent studies showed that the loss of FABP3 improves lipopolysaccharide-induced inflammation and endothelial dysfunction, also supporting our findings (53). Indeed, we found that PGPC inhibited both GSH and GPX4 expression, and FABP3 silencing reversed the PGPC-inhibited GSH and GPX4 expression. It is well known that GPX4 is the core regulatory factor of ferroptosis and can suppress ferroptosis (9), and GSH is the substrate for GPX4 which catalyzes the conversion of GSH to oxidized glutathione (23, 54). Therefore, our data suggested that PGPC enhances FABP3 expression to induce ferroptosis.

CD36 has been identified as a receptor that binds oxidized phospholipids (55, 56, 57, 58). We investigated whether PGPC upregulates FABP3 to induce ferroptosis in ECs via CD36. We found that PGPC increases CD36 expression and is involved in the regulation of ferroptosis in ECs. A recent study has shown that CD36 induces lipid peroxidation and ferroptosis through its involvement in fatty acid uptake by tumor-infiltrating CD8+ T cells in the tumor microenvironment (59, 60). In addition，CD36 was found to be involved in β-amyloid-induced ferroptosis in Alzheimer’s disease (61). Moreover, previous studies have confirmed that macrophage CD36 promotes atherosclerosis (62, 63, 64, 65), and that CD36 deficiency reduces atherosclerotic lesion formation in ECs (66). These studies suggest that PGPC promotes atherosclerosis by inducing endothelial dysfunction via CD36 to upregulate FABP3 and induce ferroptosis.

E06 directly acts against oxidized palmitoyl arachidonyl phosphatidylcholine IgM natural antibody (2, 29). A previous study has shown that E06 counteracts the action of proatherogenic oxidized phospholipids (2, 28). Low-density lipoprotein receptor null-E06-scFv mice have less atherosclerosis when fed with a Western diet (2). We investigated whether the E06 antibody could inhibit PGPC-induced ferroptosis. In the present study, we found that E06 inhibited PGPC-induced ferroptosis in ECs. Our data demonstrate that E06 is a therapeutic agent for inhibiting PGPC- or oxidized phospholipid-induced ferroptosis.

*N*-acetylcysteine, an antioxidant that acts directly on oxygen free radicals, in this study, NAC reduced the production of oxygen free radicals caused by PGPC, suggesting that PGPC can indeed induce the production of oxygen free radicals in ECs. Additionally, studies have shown that NAC treatment for 6 months, significantly slowed the progression of atherosclerosis but did not reverse atherosclerotic lesions in aging *LDLr*^*−/−*^ mice on a normal diet, despite the fact that the effect of antioxidants on atherosclerosis is inconsistent and occasionally contentious (67). More studies have also shown that NAC can reduce atherosclerosis (68, 69, 70).

**Fig. 9:**
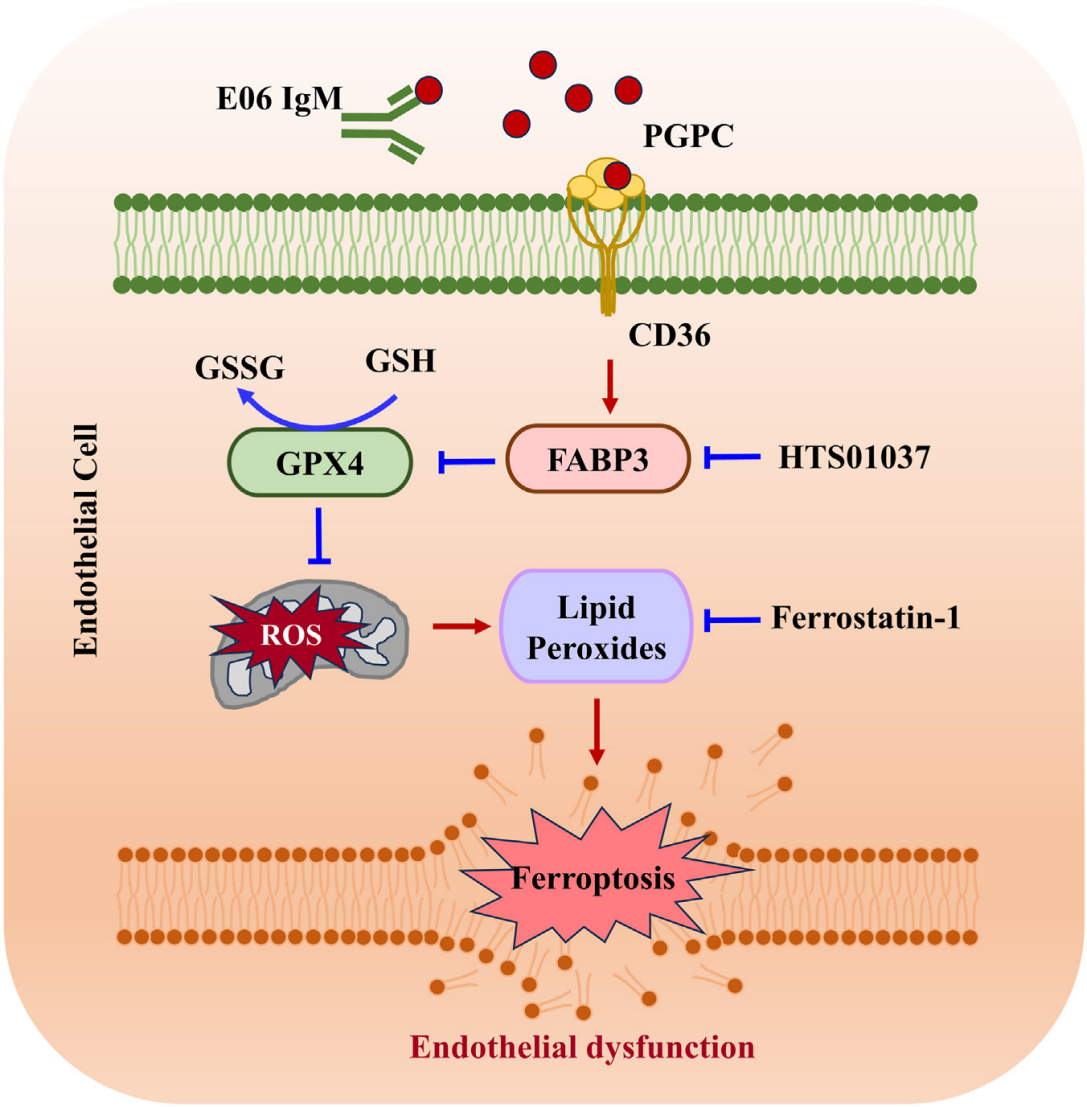
Schematic of the PGPC regulatory mechanism for ferroptosis in HUVECs.

In summary, the oxidized phospholipid component PGPC can increase FABP3 expression via the CD36 receptor to induce EC ferroptosis, resulting in endothelial dysfunction. Inhibition of FABP3 or ferroptosis can restore PGPC-impaired endothelial dysfunction. E06 also inhibits PGPC-induced EC ferroptosis Figure 9. Our findings provide new insights into the mechanisms of atherosclerosis and suggest a therapeutic approach for atherosclerosis.

## Data availability

All data are contained within the manuscript.

## Supplemental data

This article contains supplemental data.

## Conflict of interest

The authors declare that they have no conflicts of interest with the contents of this article.
