# Supplementary material for: The oxidized phospholipid PGPC impairs endothelial function by promoting endothelial cell ferroptosis via FABP3
Source: J Lipid Res. 2024 Jan 11;65(2):100499. doi: 10.1016/j.jlr.2024.100499 (PMC10864338; doi:10.1016/j.jlr.2024.100499)
Supplement: Supplemental Figures S1 and S2 [file mmc1.docx]

**The** **oxidized phospholipid PGPC impairs endothelial function bypromoting endothelial cellferroptosisvia fatty acid binding protein 3**


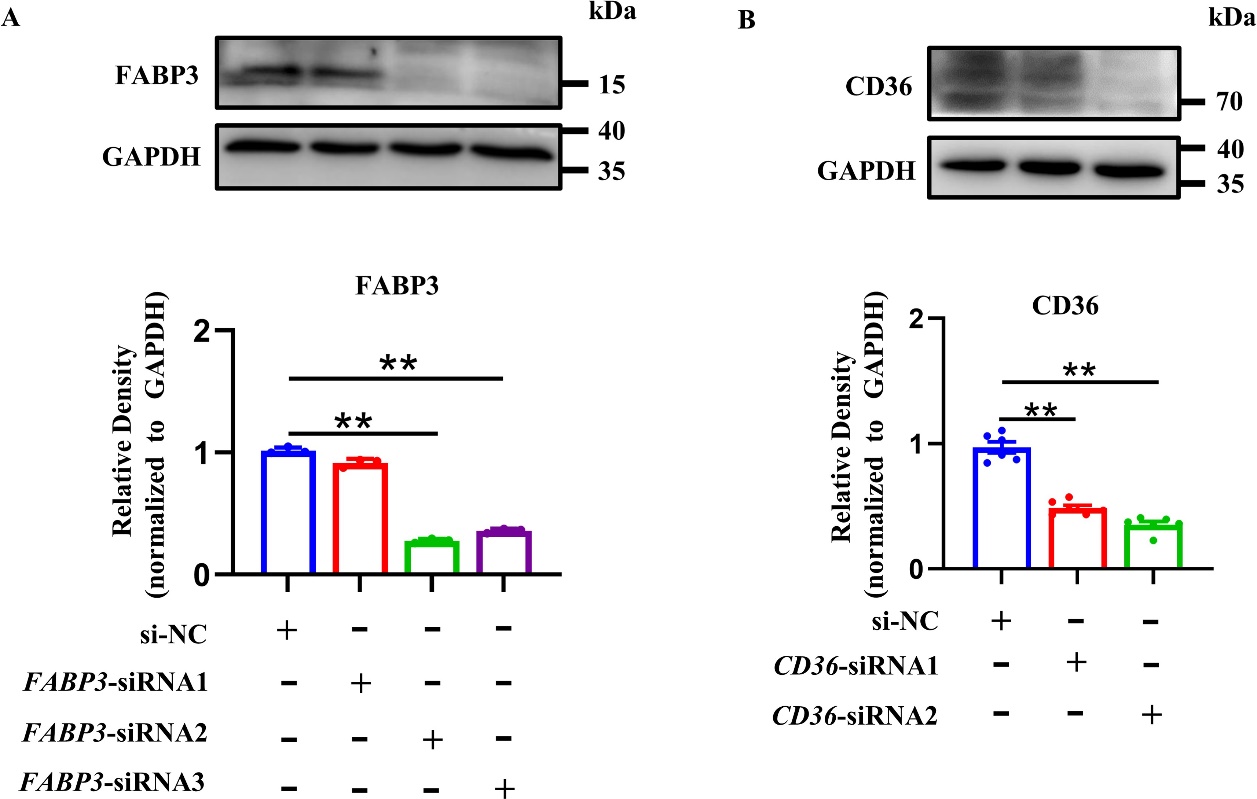


**Supplemental Figure S1**. **Fatty acid binding protein-3 (FABP3) and CD36 knockdown efficacy**

**A:** Western blots and bar charts showing the efficacy of FABP3 knockdown in human umbilical vein endothelial cells (HUVECs). FABP3-siRNAs 2 and 3 were very efficient in knockdown FABP3. **B:** Western blots and bar chart showing the efficacy of CD36 knockdown in HUVECs. CD36-siRNAs 2 was efficient in knockdown CD36. (***p*<0.01, n=3).


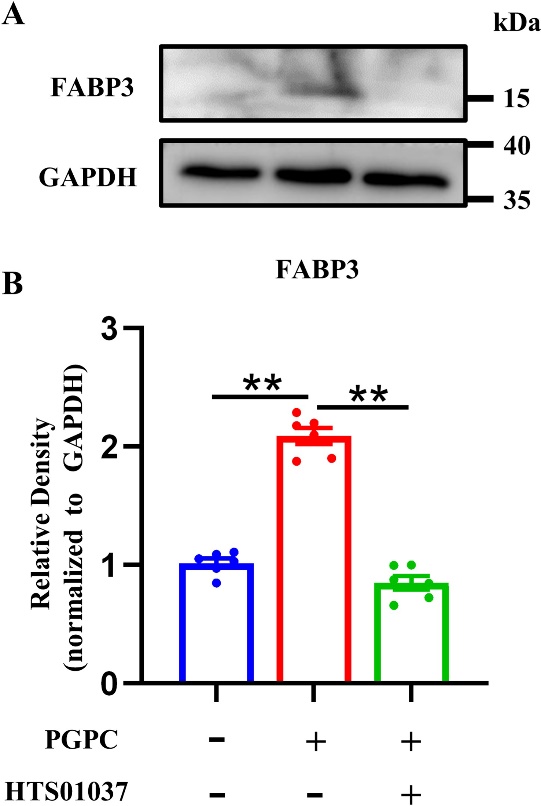


**Supplemental Figure S2. Verification of inhibitory effect of HTS01037 (FABP inhibitor) on fatty acid binding protein-3 (FABP3) in human umbilical vein endothelial cells (HUVECs)**

**A-B:** Western blots and bar chart showing that PGPC upregulates FABP3, and HTS01037 downregulates FABP3 in HUVECs. (***p*<0.01, n=6).
